# Pneumonia after Solid Organ Transplantation

**DOI:** 10.1055/a-2708-4873

**Published:** 2025-10-17

**Authors:** Paula O. Narvaez-Ramirez, Ingrid G. Bustos, Cristian C. Serrano-Mayorga, Luis F. Reyes

**Affiliations:** 1 Critical Care Department, Clínica Universidad de La Sabana, Chía, Colombia; 2 Unisabana Center for Translational Science, Universidad de La Sabana, Chía, Colombia; 3 Doctorado en Biociencias, Facultad de Ingeniería Universidad de La Sabana, Chía, Colombia; 4 Infectious Diseases, Facultad de Medicina, Universidad de La Sabana, Chía, Colombia; 5 ISARIC, Pandemic Sciences Institute, University of Oxford, Oxford, United Kingdom

**Keywords:** solid organ transplant, pneumonia, immunosuppressive therapy, infection risk

## Abstract

Solid organ transplantation (SOT) has significantly increased over the past few decades, with more than 170,000 SOTs performed worldwide in 2023. Although immunosuppressive treatments have improved patient survival, they have also increased the risk of infections among SOT recipients (SOTRs), especially pneumonia. Pneumonia remains one of the leading causes of morbidity and mortality, with respiratory infections contributing to 30 to 70% of deaths in SOTRs, depending on the organ transplanted and the timing of infection. This review summarizes current knowledge on the epidemiology, risk factors, microbial etiology, and clinical manifestations of pneumonia in SOTRs. Temporal patterns of infection are also explored, with early posttransplant infections frequently caused by nosocomial or donor-derived pathogens, and community-acquired infections predominating beyond 6 to 12 months posttransplantation. The lack of robust, SOT-specific guidelines for pneumonia complicates the management of this entity in SOTRs. Most recommendations are based on extrapolations from immunocompetent populations. Furthermore, the lack of large, prospective trials comparing empirical antibiotic strategies in SOTRs limits evidence-based decision-making. Despite these challenges, early initiation of empirical therapy remains crucial to improving outcomes. The review highlights the importance of timely microbiological diagnosis, individualized antimicrobial stewardship, and targeted therapeutic approaches in the context of increasing antimicrobial resistance. Incorporating local epidemiological data and patient-specific risk profiles may enhance the accuracy of diagnosis and support the de-escalation of therapy upon pathogen identification.

Since the first successful kidney transplant in 1954, 1 the rate of solid organ transplantation (SOT) has progressively increased, from 6.28 per million population in 2000 to 28.87 per million in 2023, with over 170.000 SOTs performed globally. Kidney transplants remain the most frequent, accounting for over 18 per million population, followed by liver, heart, and lung transplants, which together account for more than 95% of all transplants. 2 3 In 2024, the United Network for Organ Sharing reported a total of 48,149 SOTs performed only in the United States, averaging approximately 132 transplants per day. 4

Long-term immunosuppressive strategies, which have significantly reduced rates of allograft rejection and improved 1- and 5-year survival rates, have contributed to the overall success of these interventions. 5 6 However, they impose a challenge, as they place the patient at increased risk for life-threatening infections. Up to 80% of patients develop infections within the first year posttransplant, most frequently caused by bacterial pathogens (>60% throughout the first year after a SOT), followed by fungal and viral infections. Respiratory infections are the most prevalent clinically relevant infections diagnosed after lung, heart, and kidney transplants, 7 8 9 and remain among the leading causes of morbidity and mortality in SOT recipients (SOTR), accounting for 30 to 70% of deaths, depending on the site of acquisition, transplanted organ, and timeline after transplantation. 9 10 11 12 13 14 Furthermore, the development of pneumonia significantly decreases 1- and 5-year survival rates and increases the rate of organ rejection, particularly in lung transplant recipients. 9 15 Stratification by organ type and infection site reveals that reported mortality from hospital-acquired pneumonia (HAP) varies considerably across transplant populations. In lung transplant recipients, mortality ranges from 10 to 40%; in liver transplant recipients, from 22 to 50% 12 16 17 18 ; and in heart transplant recipients, from 14.7 to 30.8%. 19 20 In contrast, mortality rates in SOTR developing community-acquired pneumonia (CAP) are lower, ranging from 8 to 25% 15 21 22

## Time from Transplant to Infection

Standardized immunosuppressive regimens have enabled clinicians to identify three temporal patterns of infection based on the time elapsed since transplantation: (1) from the perioperative period to 30 days posttransplantation, during which nosocomial and donor-derived infections predominate; (2) the period from 1 to 6 to 12 months posttransplant marked by the effects of intense immunosuppression (including induction therapy and prophylaxis) and associated with opportunistic infections; and (3) beyond 6 to 12 months posttransplant period, when community-acquired infections become more prevalent. 7 23 Various prospective cohorts have aimed to determine the incidence of infection within these temporal patterns, 10 reporting a heightened burden of infection during the first year following SOT. Estimates suggest an incidence of 8.3 to 12.0 episodes per 1,000 transplant-days in the first month, 3.9 episodes per 1,000 transplant-days between 1 and 6 months, and 0.4 to 2 episodes per 1,000 transplant-days between 6 and 12 months. 7 24 25

Notably, 47% of late infections require hospitalization, underscoring their severity. 7 The site of pneumonia acquisition, CAP or HAP, is critical for assessing the temporal context of infection in SOTR. HAP and ventilator-associated pneumonia (VAP) are frequently diagnosed in the early posttransplant period, while CAP becomes more prevalent later following transplant (>6–12 months). 8

## HAP in the SOT Recipient

The type of organ transplanted significantly influences the incidence, timing, and microbiologic spectrum of nosocomial pneumonia in SOTR. 26 Lung transplant recipients are at a higher risk of pneumonia, with an incidence of up to 30%, compared with recipients of other solid organs. 7 9 27 This increased risk is not only due to the generalized immunosuppressive state but also the compromised cough reflex, impaired mucociliary clearance, disrupted lymphatic drainage, gastroparesis, prolonged mechanical ventilation, and the denervated lung allograft, all of which contribute to the heightened susceptibility. 28 29 30 31 This subgroup of SOT patients implies a significant challenge given the potential for both donor-derived infection and recipient airway colonization, which directly impact the development of pneumonia. 28 In contrast, recipients of other organs experience a lower incidence of nosocomial pneumonia, reported between 2.8 and 20% in liver transplant recipients, 11 16 32 5 to 15% in heart transplant recipients, 32 33 and 1 to 16% in Kidney transplant recipients, with increasing rates in older populations. 32 34

## CAP in the SOT Recipient

The improvement of infection prophylaxis strategies, a broader array of diagnostic tools, and enhanced immunosuppressive regimens have contributed to prolonged survival in SOTR. Consequently, CAP in these patients has become more widely understood. 8 15 23 Up to 40.7% of pneumonia cases in SOTR are diagnosed as CAP, 8 with significantly higher odds of 1-year mortality compared with non-SOTR. 22 Kidney and lung transplant recipients have a higher risk of developing CAP when compared with other SOTR. 15 35 Patients with CAP in the SOT setting tend to be younger but have a significantly higher burden of chronic comorbid conditions, particularly cardiovascular disease and chronic kidney disease, which are the most prevalent. 9 15 22 CAP episodes tend to occur earlier after transplantation in lung transplant recipients compared with liver and kidney recipients. They are more frequently preceded by therapy for acute and chronic rejection. 15 The risk of CAP due to Methicillin-resistant Staphylococcus aureus (MRSA) and Pseudomonas spp. in SOTRs appears comparable to that observed in the general population. 15 19 21

## Infection Risk Stratification in the SOT Patient

Given the broad differential diagnosis and lack of reliable assays to accurately assess the risk of infection, the clinician's evaluation of the recipient's risk becomes paramount. Fishman and Rubin, pioneers in the field of Transplant Infectious Disease, have spearheaded a comprehensive framework for assessing the recipient's infection risk. This approach defines the risk as a function of two key factors: (1) the exposures of the patient and the organ donor, including recent, nosocomial, and remote exposures, and (2) the patient's “net state of immunosuppression,” including all factors contributing to the risk of infection. 23

The term “net immunosuppression state” refers to all factors that contribute to a patient's overall risk of infection. 23 These include current immunosuppressive therapy, its timing, dosage, and individual immunological effects in patients, 36 the use of broad-spectrum antibiotics, prolonged intubation, posttransplant malnutrition, diabetes, neutropenia, infections with immunomodulating viruses (e.g., cytomegalovirus [CMV], Epstein–Barr virus, Hepatitis B and C viruses, and HIV), and genetic polymorphisms. The combined synergistic effect of these factors is not quantifiable. 37 To reduce the complexity of the above interactions, clinicians have attempted to establish an “immunological monitoring” strategy that enables the objective measurement of the overall immune response function and, consequently, the risk of posttransplant infection. An ideal tool simultaneously detects overimmunosuppression and underimmunosuppression, is highly standardized, and is straightforward to implement into routine practice. 38 A broad range of agents have been proposed in the past 20 years, using both pathogen-specific and nonpathogen-specific immune biomarkers and components of all arms of the immunologic host defense (e.g., humoral, cellular, phagocytic, and complement system) and pathogen. 36 37 38 Although notable progress has been made in posttransplant immune monitoring, its role in daily clinical practice remains unclear. Limitations of existing studies—such as small sample sizes, heterogeneous patient profiles, and imprecise characterization of infectious syndromes—are particularly evident. Future research should target well-defined SOT subgroups and establish pathogen- and graft-specific cutoffs, recognizing that immune function and infection risk are dynamic and may require repeated assessments over time.

## Etiology

The differential etiology of pneumonia in solid organ transplant SOTR is influenced by a wide range of nonspecific factors, including the recipient's net state of immunosuppression, the type of organ transplanted, the site of acquisition, environmental exposures, local epidemiology, and predisposing risk factors. 9 15 39 40 When a microbiological diagnosis is established, bacterial pathogens are most commonly identified, followed by fungal and viral etiologies. Community-acquired, hospital-acquired, or donor-derived pathogens can cause bacterial pneumonia in the SOTR. Gram-negative organisms account for the majority of HAP/VAP cases. Pseudomonas aeruginosa, Enterobacteriaceae, and S. aureus are the most frequent pathogens, with increasing rates of antimicrobial resistance over time. 8 9 12 27 29 31 39 Profiles are associated with significantly increased mortality rates and prolonged hospital stays. 41 42 43 44 45 46 In contrast, CAP bacterial isolates often consist of the widely recognized “core pneumonia bacteria,” including Streptococcus pneumoniae, Haemophilus influenzae, Moraxella spp., and S. aureus. 47 Bacterial opportunistic infections, though less frequent, can also be significant pathogens in SOTRs. These include Nocardia spp., 48 Chlamydia spp., Stenotrophomonas spp., as well as tuberculous and nontuberculous mycobacteria, 49 50 which should be considered in the diagnostic framework.

SOTRs are at increased risk of fungal infections, with the most isolated agents being Pneumocystis jirovecii, Aspergillus spp., Cryptococcus spp., Histoplasma spp., and Coccidioides spp. 46 51 Since the systematic implementation of standardized prophylaxis therapy against P. jirovecii pneumonia (PCP), its incidence has markedly decreased, now mainly occurring in late stages posttransplant or in outbreak-associated cases, which presents a public health concern. 23 52 53 Cryptococcosis occurs in approximately 2.8% of SOTRs, with one-third of cases limited to the lungs and disseminated infection occurring in up to 61% of cases. 54 55 Invasive aspergillosis has a high mortality rate, reaching up to 70% in this vulnerable population. Its development is influenced by several host-related factors, including the type of transplant, degree of immunosuppression, CMV coinfection, renal dysfunction, prior colonization, and both acute and chronic graft rejection. 56 57 58

Viral infections are not only a common cause of pneumonia in SOTRs but also predispose to secondary bacterial pneumonia, 59 which can increase rejection rates, morbidity, and mortality. 60 The most prominent causes of viral SOT pneumonia are CMV, 61 influenza, respiratory syncytial virus, human metapneumovirus, and parainfluenza. 62 63 64 65

## Clinical Presentation

In transplant populations, the clinical presentation of pneumonia is variable, and the spectrum of potential pathogenesis is broad. Signs and symptoms of pneumonia can be obscured by the inherent use of immunosuppressive therapies, which are also associated with lower leukocyte counts and maximum temperatures (i.e., antimetabolites). 66 As a result, patients may present with more subtle symptoms. For instance, cough may be absent in up to one-third of patients, and 60% of patients may not have purulent expectoration. 15 Likewise, fever may be lacking in up to 40% of patients, and noninfectious causes of fever are present in 22% of the patients. 23 If an unexplained fever is present in the SOTR, lung involvement should always be evaluated, even in the absence of specific respiratory symptoms. Up to 45% of this patient population does not present with typical examination findings of pneumonia, such as crackles or bronchial breathing. 15 The laboratory results should be carefully interpreted; for instance, white blood cell count can be variable, with neutrophilia common in bacterial infections and leukopenia or lymphopenia frequently seen in viral or opportunistic infections under high immunosuppression. 67 68 69 Lymphopenia, notably reduced CD4 +/CD8+ counts, is associated with severe viral and Legionella infections. Elevated CRP and procalcitonin correlate with disease severity, with PCT more specific for bacterial causes. 68 In Pneumocystis jirovecii pneumonia, high LDH, β-D-glucan, and hypoxemia are characteristic. 70 Positive respiratory cultures, especially for gram-negative bacteria, are frequent, though colonization without active infection can also occur. 71 Therefore, in SOTR patients, a high index of suspicion and a low threshold for empiric treatment are crucial. 40 72 Clinical evolution can vary from very rapid, indicative of bacterial or viral pathogens, to the subacute or chronic presentation often seen with fungal or mycobacterial infections. 73

## Diagnostic Approach

### Definition of Pneumonia in SOTRs

Although the burden of immunocompromised host pneumonia (ICHP) is well recognized, until 2023, there was no standardized or formally endorsed definition of this condition, which is a significant limitation to consistent clinical care and standardized identification of patients for targeted interventional trials. To address this critical knowledge gap, the American Thoracic Society (ATS) convened an expert workshop to develop a consensus statement on this topic. Using the Delphi method, the panel of experts defined ICHP as clinical suspicion of a lung infection, with or without compatible clinical signs and symptoms, and with radiographic evidence of a new or worsening pulmonary infiltrate. 74

Due to the exclusion of immunocompromised hosts from most prospective studies, there is limited evidence on optimal diagnostic approaches for SOTRs. Individual test performance is often defined in healthy subjects 75 76 77 or groups of subjects with various immunocompromising conditions. As such, diagnostic assessment in SOTRs should be highly individualized, considering local epidemiology, test availability, and patient-specific risk factors. 72 78

- Diagnostic imaging (i.e., chest X-ray, chest CT scan, or pulmonary echocardiography) should be performed in all SOT patients with suspected pneumonia. The decision on which patients should be referred for a CT scan rather than a conventional chest radiograph as initial evaluation has yet to be investigated. However, factors such as high acuity or the net state of immunosuppression could help guide this choice. 15 40
- The local epidemiology of respiratory pathogens, particularly the influenza virus, should be reviewed and considered in the evaluation. 40
- Isolation strategies should be assessed and implemented early in the patient evaluation process to prevent unnecessary and potentially harmful exposure to both patients and staff. 79
- Laboratory assessment: to assess the risk of toxicity and aid in diagnosis, complete blood analysis, liver function tests, and electrolyte chemistry should be obtained. 40

### Microbiological Workup

- The extent of microbiologic workup should be individualized, considering the presence of risk factors, time since SOT, and most likely pathogens. 40 78 Even when available diagnostic tests have suboptimal performance, etiological diagnosis should be attempted in all SOTRs with suspected pneumonia. 8 Thus, all patients with suspected pneumonia should have blood cultures drawn as soon as possible, preferably before the initiation of antibiotic therapy, as blood cultures taken within the first 24 hours of admission have 2.7 times higher odds of establishing an etiological diagnosis. Sputum analysis should be performed whenever possible for bacterial, mycobacterial, and fungal strains and cultures. 77 80 Positive cultures have a significant impact on clinical care and patient outcomes. 8 81
- CMV risk: the risk of CMV infection should be assessed by considering donor-recipient serostatus, CMV active prophylaxis, time since transplantation, and net state of immunosuppression. 23 Elevated plasma CMV viral loads are frequently observed in patients with CMV pneumonitis, but this finding alone is insufficient for diagnosis. In lung transplant recipients, CMV polymerase chain reaction (PCR) viral load in bronchoalveolar lavage (BAL) is a superior diagnostic tool than plasma CMV viral load. 40 78
- Influenza testing: nasopharyngeal influenza PCR testing is recommended to inform the pertinence of antiviral treatment. 82
- Multiplex molecular respiratory virus testing: multiplex molecular respiratory virus testing should be considered in the evaluation of pneumonia in SOTR, particularly during seasons with high respiratory virus incidence. Identifying a viral etiology can influence clinical decisions regarding further evaluation and the need for extended antimicrobial therapy; however, the impact of this testing has not been explicitly evaluated in SOTRs. 83 Detection of a virus by PCR does not exclude the possibility of concurrent bacterial infection, and a negative PCR does not completely rule out a viral etiology. If clinical suspicion remains high despite a negative result, a more invasive approach—such as bronchoscopy with lower respiratory tract sampling—should be considered. 78 84 Urinary antigen testing for S. pneumoniae, Legionella pneumophila, and Histoplasma capsulatum should be considered, especially in the setting of disseminated disease. 85 86

### Invasive Identification Strategies

BAL, transbronchial biopsy, and lung biopsy may be considered in selected patients. BAL can yield an etiologic diagnosis in approximately 39 to 77% of patients with SOT pneumonia. 21 The decision to perform bronchoscopy or BAL should be individualized. 40 87 In this patient subset, maintaining a low threshold for invasive diagnostic strategies is advisable, particularly in the presence of clinical instability, lack of improvement with empiric antibacterial therapy when a diagnosis has not been established through noninvasive testing, and the presence of diffuse or focal pulmonary infiltrates. 40 78 88 89 The specific tests performed on BAL are not standardized and should be guided by clinical presentation, imaging findings, and immunologic risk factors. 40 78 The following array of tests might be considered:

- Bacterial, fungal, viral, and mycobacterial culture. 78
- Diagnostic testing, including PCP-directed stains or nucleic acid-based testing for PCP from BAL fluid. 90
- Nucleic acid-based testing (PCR, QNAT, Film array) for respiratory viruses, CMV, MRSA, Mycoplasma spp, Ureaplasma spp, and/or Legionella spp, Nocardia spp., Mycobacterium tuberculosis, depending on test availability and the patient's clinical history. 84 88 91
- Galactomannan in patients with findings suggestive of invasive fungal infection. 78 92 93

**Fig. 1:**
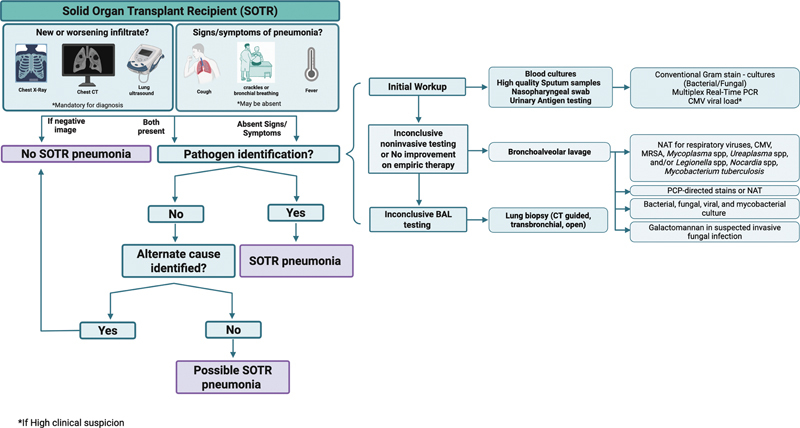
Definition and stepwise microbiologic workup of pneumonia in the solid organ transplant recipient (SOTR). The algorithm is triggered when a SOTR has respiratory signs/symptoms and/or a new or worsening infiltrate. Confirmation of pneumonia requires imaging evidence (symptoms may be absent). Pathogen identification then guides classification as confirmed or possible SOTR pneumonia, with alternate diagnoses considered. The microbiologic workup progresses from noninvasive tests to BAL and, if needed, lung biopsy, including bacterial, fungal, viral, and mycobacterial cultures, nucleic acid tests, and targeted assays such as galactomannan. BAL, bronchoalveolar lavage; CMV, cytomegalovirus; CT, computed tomography; MRSA, methicillin-resistant Staphylococcus aureus; NAT, nucleic acid test; PCP, Pneumocystis jirovecii pneumonia; SOTR, solid organ transplant recipient. Created in BioRender. Universidad de la sabana, C. [2025] https://BioRender.com/3yaerhf.

Lung biopsy has demonstrated an etiologic diagnostic yield of approximately 85%, but with a high complication rate. 94 CT-guided lung biopsy, using either fine-needle aspiration or core biopsy, is associated with lower complication rates, although it comes at the expense of reduced diagnostic accuracy. 95 A thorough risk–benefit analysis should be conducted before selecting this diagnostic approach. Current guidelines recommend lung biopsy for patients who do not achieve clinical stability and in whom BAL testing fails to establish a diagnosis, as well as for patients with focal pulmonary nodules where diagnosis has not been determined by other methods, given the potential risk for malignancy or invasive fungal disease. 78 The definition and sequential diagnostic strategy for pneumonia in the SOTR, as described above, is illustrated in Fig. 1 .

## Treatment

Given the complex nature and significant determinants of pneumonia etiology in SOTRs, such as the net immunosuppressive state, time since transplant, and the pace of illness development, along with the lack of robust trials systematically endorsing specific clinical practices, guidelines for the management of CAP and HAP/VAP systematically exclude this patient population from their recommendations. 75 76 77 80 96 Furthermore, the heterogeneity in definitions of immunosuppression across studies and guidelines limits the standardization and comparability of results, making it challenging to apply evidence uniformly. Another challenge is that most studies evaluate outcomes of antimicrobial therapy in patients whose pneumonia etiology has been identified. No extensive prospective studies comparing different empirical therapies in SOTRs have been conducted. In response to this gap, the American Society of Transplantation Infectious Diseases (AST ID) Community of Practice published a set of recommendations in 2019, focusing on the treatment of pneumonia in SOTR, based on the available evidence and expert clinical experience. 40 Since the timely initiation of appropriate empirical therapy is independently associated with hospital mortality, 97 it is crucial that SOT pneumonia management prioritizes the correct initiation of treatment, which should consider the following ( Table 1 ):

**Table 1:** Considerations before initiating empiric antibiotic therapy in solid organ transplant recipients

1	Previously documented microbial colonization
2	Prior antimicrobial resistance patterns of colonizing or pathogenic organisms
3	Local epidemiology of resistance patterns
4	Current immunosuppressive regimen
5	Potential drug–drug interactions
6	Site of care (based on clinical presentation and imaging findings)

- Microbial colonization and resistance: previously known microbial colonization and prior antimicrobial resistance patterns of specific colonizing or pathogenic organisms, with particular emphasis on MDR bacteria colonizing the airway in lung transplant recipients, 9 98 the local epidemiology and rates of multi-drug-resistant pathogens, and the potential for drug interactions with immunosuppressive agents must be evaluated to ensure that antimicrobial therapy does not compromise treatment effectiveness. 99 ( Table 1 )
- Site of care: hospitalization in SOT patients with CAP is primarily based on clinical judgment and the AST ID guidelines 40 and The Consensus on the treatment of CAP in Immunocompromised Adults 78 recommend against using pneumonia severity scores (e.g., CURB-65, and Pneumonia Severity Score) to guide treatment settings, as these scores have not been well validated in SOTRs and have demonstrated poor performance in other immunosuppressed host populations. 100 101 Moreover, the largest academic transplant center in Germany assessed the performance of seven risk scores for predicting prognosis and in-hospital mortality among SOTR patients with CAP, finding that none exhibited high discriminative power. 102 Blood oxygen saturation and chest CT findings might assist in the decision regarding hospitalization. 78 103 Patients may present with subtle symptoms at onset but can rapidly deteriorate; therefore, a low threshold for hospitalization is advised. If outpatient care is considered, close follow-up mechanisms should be in place. 78
- Empiric therapy for hospitalized patients: in patients requiring hospitalization, combination therapy with a β-lactam (± MRSA and ± antipseudomonal activity) plus an agent active against intracellular organisms (Mycoplasma spp. and Legionella spp.) is recommended. The breadth of coverage of the penicillin-based agent used depends on local resistance patterns (especially for S. pneumoniae) and the presence of risk factors, such as previous colonization, recent hospitalization, structural lung disease, or the presence of prosthetic material, which warrants coverage against hospital-acquired gram-negative pathogens. Fluoroquinolones should be considered as an alternative in this setting. 40
- Inpatients hospitalized for CAP who test positive for a respiratory virus should receive a prescription of empiric antibiotics, as the high risk of poor outcomes with viral-bacterial coinfection is particularly pronounced when antibiotic therapy is delayed or withheld. 96 104 105
- Empiric therapy for severe CAP: the presence of severe CAP may be used as an indication to start empirical therapy for resistant gram-positive and gram-negative organisms, followed by rapid de-escalation if no multidrug-resistant pathogen is identified. Although the severity of CAP is not an accurate predictor of drug resistance or opportunistic infections, inadequate initiation of early empiric treatment is independently associated with increased mortality; thus, severe pneumonia or pneumonia requiring ICU care can be used as a threshold to start empirical therapy for resistant organisms (e.g., MRSA and Pseudomonas spp.). 78
- When suspected HAP/VAP, international/national guidelines apply, with attention to local guidelines, prevalence, and epidemiology. Hence, empirical antibiotic therapy should include coverage against P. aeruginosa, other Gram-negative bacilli, and S. aureus. 40 41 77 106 107
- Outpatient management: for outpatient management in patients with a low risk for opportunistic or hospital-acquired infections, Beta-lactams, macrolides, or fluoroquinolones (especially in settings with a high incidence of macrolide resistance) with coverage of respiratory pathogens may be considered for empiric therapy. A crucial consideration in this context is the potential for drug interactions between macrolides and immunosuppressive agents. 40 The inclusion of an agent active against MRSA or Pseudomonas spp. is dependent on local resistance patterns, prevalence, the individual patient's infection, and bacterial colonization history, as well as individual features of the presenting illness. 80 108 109
- Duration of antibiotic therapy: the optimal duration of antibiotic treatment remains uncertain in both immunocompromised and immunocompetent patients. Given the negative effects of antibiotic use on the microbiome—including reduced species diversity, selection of resistant bacteria, and altered metabolic activity—the primary goal of therapy may no longer be complete eradication of the causative pathogen, but rather reduction of the bacterial load with minimal disruption of the microbiome. Current general guidelines suggest at least a 5-day antibiotic course. Clinical stability parameters (i.e., temperature ≤ 37.8°C, heart rate < 100 beats per minute, < 24 breaths per minute, SpO _2_ ≥ 90% or PaO _2_ ≥ 60 mm Hg on room air or baseline oxygen requirement, systolic blood pressure > 90, and a normal mental status) are decisive in guiding antibiotic duration, 96 as well as laboratory improvement (e.g., procalcitonin guided reduction of antibiotic therapy in sCAP patients 76 and microbiological eradication can further guide the decision to stop therapy). 40 77 Recently published ATS/IDSA CAP guidelines recommend less than 5 days (at least 3 days, rather than 5 or more) for outpatients and inpatients who reach clinical stability; however, these recommendations are based exclusively on randomized controlled trials in immunocompetent patients and may not be directly applicable to SOTRs.
- Empiric antiviral therapy: all patients presenting with influenza-like symptoms benefit from empiric administration of an antiviral drug active against the virus (i.e., neuraminidase inhibitors such as oseltamivir or zanamivir) before microbiological confirmation, preferably within the first 48 hours of symptom onset, especially during influenza season. 72 Although these drugs have not been specifically tested in clinical trials involving SOT populations diagnosed with influenza, available data demonstrate that early initiation (<48 hours) of oseltamivir or zanamivir is associated with decreased mortality, reduced ICU admissions, 82 110 111 112 113 and decreased allograft dysfunction, particularly in lung transplant recipients. 112 In Kidney transplant recipients, baloxavir marboxil (an inhibitor of cap-dependent endonuclease approved as treatment of uncomplicated influenza in immunocompetent patients) 114 appears to be as effective as oseltamivir for treating influenza when compared with oseltamivir and was associated with a faster symptom resolution when given after 48 hours of symptom onset. 115
- Antiviral therapy duration: duration of antiviral therapy should last at least 5 days; prolonged courses of treatment may be considered upon persistence of symptoms. 72
- Empiric therapy for opportunistic pathogens: empiric therapy against Nocardia spp., tuberculosis, nontuberculous mycobacteria, endemic fungi, and invasive molds is typically not instituted before diagnostic testing or a procedure. Practitioners taking care of patients with subacute or chronic illness presentations that do not improve with suggested regimens should consider these pathogens in their differential diagnosis. 40
- Extended empirical antimicrobial coverage: with the above considered, experts on pneumonia in immunocompromised patients suggest extended empirical antimicrobial coverage in the following scenarios 78 :
  ○ PCP: initial empirical therapy with trimethoprim-sulfamethoxazole (TMP/SMX) should be extended to cover the possibility of PCP pneumonia in patients with diffuse, bilateral, interstitial infiltrates or alveolar opacities and who are not receiving PCP prophylaxis (according to indication) or in non-HIV hosts with severely impaired cell-mediated immunity (e.g., taking glucocorticoids with cytotoxic agents).
  ○ Nocardia spp.: empirical therapy with TMP/SMX for the possibility of pneumonia caused by Nocardia spp. should be given to patients with heart, lung, or liver transplant with a lung abscess who have not been receiving adequate prophylaxis with TMP-SMX. If TMP/SMX is contraindicated, linezolid can be considered.
  ○ CMV: addition of empirical therapy with ganciclovir may be considered in patients with bilateral interstitial pneumonia after a recent lung transplant.

As the appropriateness of initial empiric therapy is measured in large part by the patient's clinical response, close monitoring is warranted, especially during the first 24 to 48 hours of treatment. All empiric therapies should be de-escalated upon microbiological identification. A summary of the empiric treatment recommendations is described in Table 2 .

**Table 2:** Empiric antimicrobial therapy on solid organ transplant recipients according to site of care

Site of care	Empiric therapy recommendations
Outpatient	Beta-lactams, macrolides, or fluoroquinolones (fluoroquinolones > macrolides in high macrolide resistance therapy)
	Consider drug–drug interactions between macrolides and immunosuppressive agents
	Include anti-MRSA/Pseudomonas coverage according to local epidemiology and the presence of risk factors
Inpatient (nonsevere CAP)	Combination therapy with a beta-lactam (± MRSA, ± antipseudomonal coverage) plus an intracellular-active agent (e.g., macrolide or fluoroquinolone for Mycoplasma spp., Legionella spp.)
	If positive viral identification, patients should still receive empiric antimicrobials due to increased risk of bacterial coinfection
Severe CAP/ICU admission	Include anti-MRSA/Pseudomonas coverage until pathogen identification is available
	Antimicrobial de-escalation should follow if no multidrug-resistant pathogens are identified
Opportunistic/extended coverage	PCP: TMP/SMX in patients with diffuse bilateral interstitial infiltrates not on prophylaxis, or non-HIV immunocompromised hosts with impaired cellular immunity
	Nocardia spp.: TMP/SMX (or linezolid if contraindicated) in transplant recipients with lung abscesses without prophylaxis
	CMV: empiric ganciclovir in recent lung transplant patients with bilateral interstitial pneumonia
Antiviral empiric therapy	Empiric neuraminidase inhibitors (e.g., oseltamivir and zanamivir) for influenza-like illness, ideally within 48 h of symptom onset

Abbreviations: CMV, cytomegalovirus; MRSA, methicillin-resistant Staphylococcus aureus; PCP, Pneumocystis jirovecii pneumonia; TMP/SMX, trimethoprim/sulfamethoxazole.

## Future Directions for Research

The management of pneumonia in SOTRs remains challenging due to the lack of comprehensive prospective studies specifically addressing microbiological diagnosis and empirical treatment. Current guidelines often lack SOT-specific data, relying instead on studies involving immunocompetent populations. Research should focus on optimizing diagnostic methods, especially molecular techniques, to enable rapid pathogen identification and guide timely, appropriate therapy. Additionally, studies comparing different antimicrobial regimens, accounting for resistance patterns, timing of infections, and organ type, will help tailor treatments to SOTRs' specific needs. Personalized approaches, balancing immune suppression to prevent rejection while minimizing infection risks, must be prioritized. Finally, long-term follow-up studies assessing the effectiveness of prophylactic strategies and de-escalation protocols in reducing pneumonia-related mortality in SOTRs will significantly aid in advancing patient care.
